# Liquid-Microjet Photoelectron Spectroscopy of the Photoactive Yellow Protein Chromophore in Aqueous Solution

**DOI:** 10.1021/acs.jpca.6c02627

**Published:** 2026-06-12

**Authors:** Edoardo Simonetti, Anton N. Boichenko, Johanna Rademacher, Alice Henley, Kate Robertson, Harmanjot Kaur, Sebastian Malerz, Iain Wilkinson, Bernd Winter, Anastasia V. Bochenkova, Helen H. Fielding

**Affiliations:** † Department of Chemistry, 4919 University College London, 20 Gordon Street, London WC1H 0AJ, U.K.; ‡ Department of Chemistry, 64935 Lomonosov Moscow State University, Moscow 119991, Russia; § Molecular Physics Department, 28259 Fritz-Haber-Institut der Max-Planck-Gesellschaft, Berlin 14195, Germany; ∥ Institute for Electronic Structure Dynamics, 28340 Helmholtz-Zentrum Berlin für Materialien und Energie, Berlin 14109, Germany

## Abstract

Photoactive yellow protein (PYP), a prototypical photoreceptor responsible for the photophobic response of the *Halorhodospira halophila* bacterium to harmful ultraviolet (UV) radiation, is known to undergo photooxidation in aqueous solution. However, the vertical detachment energy and electronic structure of the deprotonated chromophore that lies at the heart of PYP have not been measured in aqueous solution. Here, we use X-ray, extreme ultraviolet (EUV), and multiphoton UV liquid-microjet photoelectron spectroscopy, supported by high-level quantum chemistry calculations, to map out the electronic structure of the deprotonated PYP chromophore in aqueous solution. The vertical and adiabatic electron detachment energies are found to be 6.8 ± 0.1 eV and around 5.9 eV, respectively. Multiphoton UV photoelectron spectroscopy measurements confirm the existence of a high-lying two-photon resonance close to the detachment threshold that could be responsible for UV photooxidation, and they reveal the existence of a three-photon resonance in the detachment continuum. This work demonstrates the power of combining X-ray, EUV, and UV liquid-microjet photoelectron spectroscopy to unravel the electronic structure of weakly soluble organic chromophores, paving the way for deeper insights into their roles in photobiological processes.

## Graphical abstract

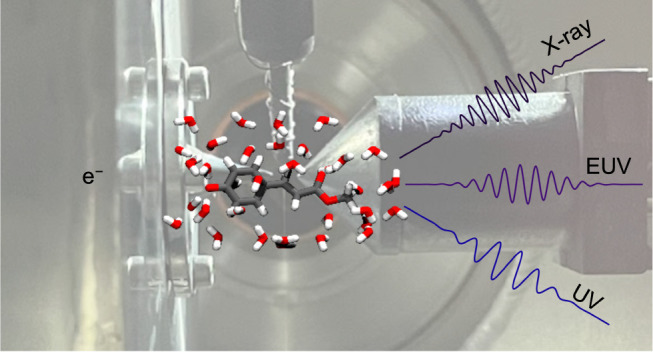

## Introduction

Photoactive proteins are ubiquitous in biological systems and often control the response of living organisms to light in their environment. Photoactive yellow protein (PYP) is responsible for the negative phototaxis of the *Halorhodospira halophila* bacterium and has been studied extensively as a prototypical photoreceptor.^1^ At the heart of PYP is its chromophore, deprotonated *trans*-*p*-coumaric acid, which is bound to a cysteine residue via a thioester link and stabilized by a network of hydrogen bonds. The photocycle of PYP has been investigated using several experimental techniques including X-ray crystallography,^2–5^ optical transient absorption^6–10^ and infrared spectroscopy.^11–13^ It is initiated by the absorption of a photon of blue light (λ_max_ = 446 nm corresponding to 2.78 eV) and the consequent *trans*-*cis* isomerization of the C=C double bond of the chromophore induces a cascade of structural changes of the protein to signal the presence of harmful UV radiation. A clear picture of the electronic structure of the chromophore and how complex environments can tune its photoresponse is essential to obtain a comprehensive understanding of the isomerization process involved in the photocycle.

Gas-phase anion PES studies of the impact of chemical and structural modifications of the isolated PYP chromophore have highlighted the role of substituent groups at the carbonyl tail of the PYP chromophore on the electronic structure and excited-state dynamics.^14–22^ It was found that chromophores with stronger electron-accepting groups have higher vertical detachment energies (VDEs), due to increased stabilizing effects of the substituents.^15^ Studies of chromophores with locks around the double bond or adjacent single bonds, led to the understanding that rotations about both the double bond and the single bond between the double bond and the phenolate, guide the molecule toward conical intersections with the ground state.^16^

Similar observations have been obtained from time-resolved experiments of PYP chromophore analogues in aqueous solution,^23–33^ where the electron-accepting character of the substituting group at the carbonyl tail has been shown to affect the relaxation time scales and determine whether isomerization occurs. Chromophores with strong electron acceptors have been shown to relax quickly via a phenolate-twisted ground-state intermediate to the *trans* isomer, whereas chromophores with weak electron acceptors relax directly to the *cis* isomer on a slower time scale.^29,30,33^ Transient absorption spectroscopy studies have also revealed the formation of solvated electrons following excitation of PYP and PYP chromophores in aqueous solution.^7,9,10,23,26,27,29,30,32^ It was proposed that the solvated electrons were formed by a two-photon process, based on a quadratic dependence of the solvated electron absorption signal on excitation pulse energy. However, the mechanism of solvated electron formation is still debated, specifically whether it arises from a charge-transfer-to-solvent state or from a resonant intermediate in a multiphoton 1 + 1 excitation process.^10^ For PYP in aqueous solution, the diffusion of the solvated electron controls geminate recombination with the radical of the chromophore, and the diffusion coefficient determined from the geminate recombination time scale was similar to that in water.^10^ Therefore, determining the detachment energies and investigating the higher-lying electronically excited states proposed to be involved in the formation of solvated electrons in aqueous solution can provide information about the photoredox properties of this important chromophore, which can then be used as a basis for understanding similar processes *in vivo*.

Liquid-microjet photoelectron spectroscopy (LJ-PES) is one of the most powerful experimental techniques for studying the electronic structure of molecules in aqueous solution because it provides a direct measure of electron binding energies (eBEs). X-ray and extreme ultraviolet (EUV) LJ-PES are excellent methods for the determination of accurate eBEs of solute molecules in their ground electronic state as the photon energies are high enough to generate photoelectrons with electron kinetic energies (eKEs) ≳15 eV, which ensures that the distributions of inelastically scattered photoelectrons are well separated from the spectral features of interest.^34,35^ A challenge for X-ray and EUV LJ-PES of sparingly soluble organic chromophores is that high solute concentrations (≳5 mM) or long acquisition times are generally required to ensure a sufficient signal-to-noise ratio of the photoelectron spectrum of interest since the photoelectron spectrum of water (55.5 M) dominates. Ultraviolet (UV) LJ-PES has the advantage that multiphoton ionization/detachment can be employed for μM concentrations, with UV photons that do not have enough energy to ionize water. At these low concentrations and with low concentrations of electrolyte, aggregation and chemical reactions such as hydrolysis are less likely. Moreover, resonance-enhanced multiphoton PES can also measure eBEs of electronically excited states which determine the dynamics of processes such as charge transfer and can be difficult to measure using other methods. A challenge with UV LJ-PES has been that inelastic electron scattering is dominated by sub-eV energy losses and distorts the spectra; however, recent progress in retrieving true photoelectron spectra from such distorted spectra has made UV LJ-PES a powerful tool for unraveling the electronic structure of weakly soluble organic chromophores.^36–41^

Here, we present a combined X-ray, EUV, and multiphoton UV liquid-jet photoelectron spectroscopy study of the deprotonated methyl ester derivative of *trans*-*p*-coumaric acid (*p*CE^–^, Figure 1) in aqueous solution, to determine vertical detachment energies and estimate adiabatic detachment energies, and to explore the high-lying electronically excited states. To assist with the interpretation of our experimental measurements, we employ multistep molecular dynamics (MD) and quantum mechanics/effective-fragment potential (QM/EFP) computational chemistry calculations.

**Figure 1:**
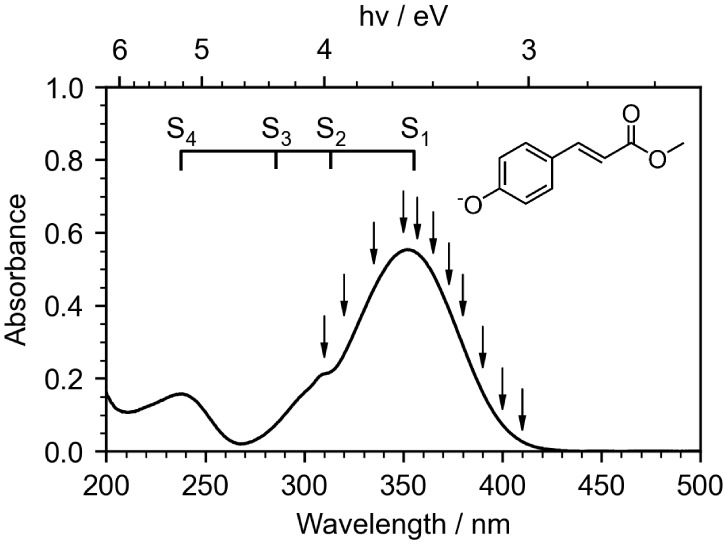
UV–vis absorption spectrum of 50 μM aqueous solution of *p*CE^–^ in a 4 mm cuvette. Arrows indicate the wavelengths employed for the UV LJ-PES measurements reported in this work and the comb indicates the vertical excitation energies of the first four electronically excited states of aqueous *p*CE^–^ calculated at the XMCQDPT2/(aug)-cc-pVDZ/EFP level of theory. Inset: structure of *p*CE^–^.

## Methods

### Experimental

Aqueous solutions of *p*CE^–^ were prepared with *p*CE (>98.0%, Tokyo Chemical Industries) and NaOH (Sigma-Aldrich), and sonicated for up to 2 h until the *p*CE^–^ was fully dissolved. In the X-ray and EUV LJ-PES measurements, NaF and NaCl were also added to the solutions, respectively, to ensure sufficient electrical conductivity of the solutions.

UV–visible steady-state absorption spectra of 50 μM *p*CE in 2.0 mM aqueous NaOH and 2.0 mM aqueous NaOH were recorded with a UV-2600i spectrophotometer (Shimadzu).

X-ray photoelectron spectra of 5.0 mM *p*CE in aqueous solution with 5.5 mM NaOH and 30 mM NaF were recorded at the U49/2 PGM-1 beamline (20 × 80 μm^2^ focal size) of BESSY II (Berlin), using a photoelectron spectrometer detailed elsewhere,^42^ which consists of a differentially pumped hemispherical electron analyzer and an interaction chamber. Solutions were cooled to 12 °C and introduced in the interaction chamber via a 25 μm diameter fused silica nozzle with an approximate velocity of 20 ms^–1^. No voltage was applied to the liquid jet, which was electrically grounded to the spectrometer. The photon energy was 180 eV and the energy resolution of the experiment was <125 meV, determined by the energy resolution of the detection scheme (∼100 meV) and of the beamline (∼100 meV).

EUV photoelectron spectra were recorded using the monochromatised He II α­(*h*ν = 40.81 eV) line of a helium discharge source (Scienta Omicron VUV5K). Experiments were performed using the EASI (Electronic structure of Aqueous Solutions and Interfaces) setup,^43^ which consists of a differentially pumped hemispherical electron analyzer and an interaction chamber housing a liquid microjet. Solutions of (i) 5.0 mM *p*CE in aqueous 5.5 mM NaOH and 50 mM NaCl, or (ii) 50 mM NaCl were maintained at 20 °C, and a −25 V voltage was applied to the solutions before being introduced in the chamber through a 26 μm diameter fused silica nozzle using a high performance liquid chromatography pump at a flow rate of 1.5 mL/min. The pressure in the interaction region was maintained between 1 × 10^–4^ and 4 × 10^–4^ mbar. The liquid jet was intersected by monochromatised radiation (∼1 meV fwhm bandwidth) originating from the helium source and photoelectrons were detected by the hemispherical analyzer in 25 meV energy bins.

UV photoelectron spectra of *p*CE^–^ were measured using a liquid-microjet magnetic-bottle time-of-flight (TOF) photoelectron spectrometer described in detail elsewhere.^44^ Aqueous solutions of 5 or 50 μM *p*CE in 1.0–3.5 mM NaOH were introduced through a 20 μm diameter fused silica nozzle using a high-performance liquid chromatography pump operating with flow rates between 0.50 and 0.95 mL/min. The concentration of NaOH and the liquid jet flow rate were varied to minimize the difference between the vacuum level at the walls of the interaction region and vacuum level at the jet, which is affected by electrokinetic charging of the liquid inside the nozzle. The liquid jet was intersected by tunable femtosecond laser pulses (410–310 nm, 4–10 nJ/pulse) around 1 mm below the orifice, before being collected in a cold trap. Photoelectrons were detected at the end of a TOF tube as a function of arrival time relative to the trigger of the laser pulse. Photoelectron spectra of NO and Xe were recorded to determine the conversion from TOF to electron kinetic energy (eKE), the energy resolution (Δ*E*/*E*∼1%) and the energy-dependent collection efficiency of the spectrometer.

To achieve a uniform vacuum level in the interaction region, we coated the liquid-jet nozzle, the skimmer, the gas nozzle and the magnet with colloidal graphite. However, during our measurements, water molecules adsorb onto the graphite thus gradually changing the work function of the surfaces around the interaction region. This effect, together with the streaming potential induced by electrokinetic charging inside the nozzle, can create a potential gradient in the interaction region. Therefore, we measured the shift in eKE of the photoelectron spectra of Xe as a function of distance of the liquid jet from the interaction point before each measurement, and adjusted the NaOH concentration and flow rate of the liquid jet to ensure a uniform vacuum level in the interaction region. We repeated the same measurement at the end of each set of LJ measurements, and observed shifts in the position of the vacuum level smaller than our experimental error (Figure S1).

NMR studies indicate negligible aggregation, but suggest that some hydrolysis occurs in the 5 mM solutions (Figures S2 and S3).

### Computational

A ground-state geometry of the solvated chromophore anion was obtained using a multistep hybrid QM/EFP/MD approach developed by us earlier.^45,46^ The system comprised a central QM region including *p*CE^–^ and five water molecules closest to the chromophore; an inner region of ∼1000 water molecules that were optimized under a QM/EFP^47,48^ description; and an outer water shell that was relaxed using MD simulations within classical TIP3P^49^ force field parameters (Figure S4). The QM part was treated at the PBE0/(aug)-cc-pVDZ level of theory, with diffuse functions only affixed to oxygen atoms. The radius of the water sphere around the solute was varied from 10 to 36 Å. The first VDE was calculated as the energy difference between the S_0_ and D_0_ states in the geometry of the anion at the PBE0/(aug)-cc-pVDZ/EFP level of theory for a series of the model systems of increasingly larger size with up to ∼10,600 water molecules. All water molecules, except for those included in the QM part, were treated as EFPs. A structure with ∼7,700 water molecules (R = 34 Å), which showed the converged D_0_ VDE value (Figure S4), was then used for the higher-level XMCQDPT2 ^50^/SA­(10)-CASSCF­(12,12)/(aug)-cc-pVDZ+/EFP calculations of D_0_, D_2_, and D_3_ (π^–1^) VDEs, with a very diffuse function placed outside the water sphere and included in the pure π valence active space to mimic electron detachment. The D_1_ (n^–1^) VDE was calculated as a sum of D_0_ VDE and D_0_ → D_1_ VEE. A smaller-sized, pure QM/EFP system was used for excited-state calculations. The vertical excitation energies in the solvated neutral radical were calculated at the XMCQDPT2/SA(7)-CASSCF­(11,11)/(aug)-cc-pVDZ/EFP using a mixed n/π active space. The vertical excitation energies of the solvated anion were calculated using both the pure and mixed active spaces at the XMCQDPT2/SA(7)-CASSCF­(12,11)/ (aug)-cc-pVDZ/EFP and XMCQDPT2/SA(7)-CASSCF­(14,12)/(aug)-cc-pVDZ/EFP levels of theory, respectively. The Firefly computational package^51^ was used for all electronic structure calculations. The MD simulations were performed with NAMD.^52^

## Results and Discussion

### UV–vis Absorption Spectrum

Figure 1 shows the UV–vis absorption spectrum 50 μM aqueous *p*CE^–^, presented as the difference between absorption spectra of 50 μM *p*CE in 2.0 mM aqueous NaOH, and 2.0 mM aqueous NaOH, in 4 mm cuvettes. The absorption maximum is 351.5 nm (3.53 eV), which is blue-shifted with respect to the absorption maxima in the protein (446 nm, 2.78 eV)^1^ and in vacuum (430 nm, 2.88 eV).^19,53^ The comb marks the first four vertical excitation energies of aqueous *p*CE^–^ calculated at the XMCQDPT2/(aug)-cc-pVDZ/EFP level of theory.

### Vertical and Adiabatic Detachment Energies

Figure 2 shows the photoelectron spectra of aqueous *p*CE^–^ recorded with photon energies of 180 eV (a, d), 40.81 eV (b, e), and 3.02 eV (c, f), plotted as a function of *nh*ν-eKE, where *n* = 1 (a, b, d, e) or *n* = 3 (c, f). The X-ray and EUV spectra in Figure 2a, b are plotted as difference spectra, i.e., the water spectral contributions were subtracted from the corresponding aqueous solution spectra, and their intensities have been normalized to the maximum of the 1b_1_ valence band of liquid water. The 180 eV difference spectrum was obtained by fitting the recorded solution and solvent spectra with a sum of Gaussians describing photodetachment from *p*CE^–^ and photoionization from liquid and gaseous water. The widths and relative central eKEs of the liquid water peaks were constrained to the values obtained from recent accurate measurements of the valence band of liquid water.^54^ The energy scale was calibrated by setting the water 1b_1_ eBE to the accurate eBE value reported in the same study. The contribution from the water peaks was subtracted from the measured spectrum yielding the difference spectrum in Figure 2a. The 40.81 eV difference spectrum was obtained by subtracting a 50 mM NaCl spectrum smoothed with a 12-point quadratic Savitzky-Golay filter from the *p*CE^–^ spectrum. These EUV spectra were recorded with a −25 V voltage applied to the jet. The energy scale of the difference spectrum was determined in two steps, using the energy position of the low-energy cutoff, which corresponds to zero kinetic energy,^54^ to calibrate a complete EUV reference spectrum, and the maximum position of the resulting water 1b_1_ peak to energetically align a higher signal-to-noise-ratio spectrum recorded over a smaller 5–11 eV detachment energy range.

**Figure 2:**
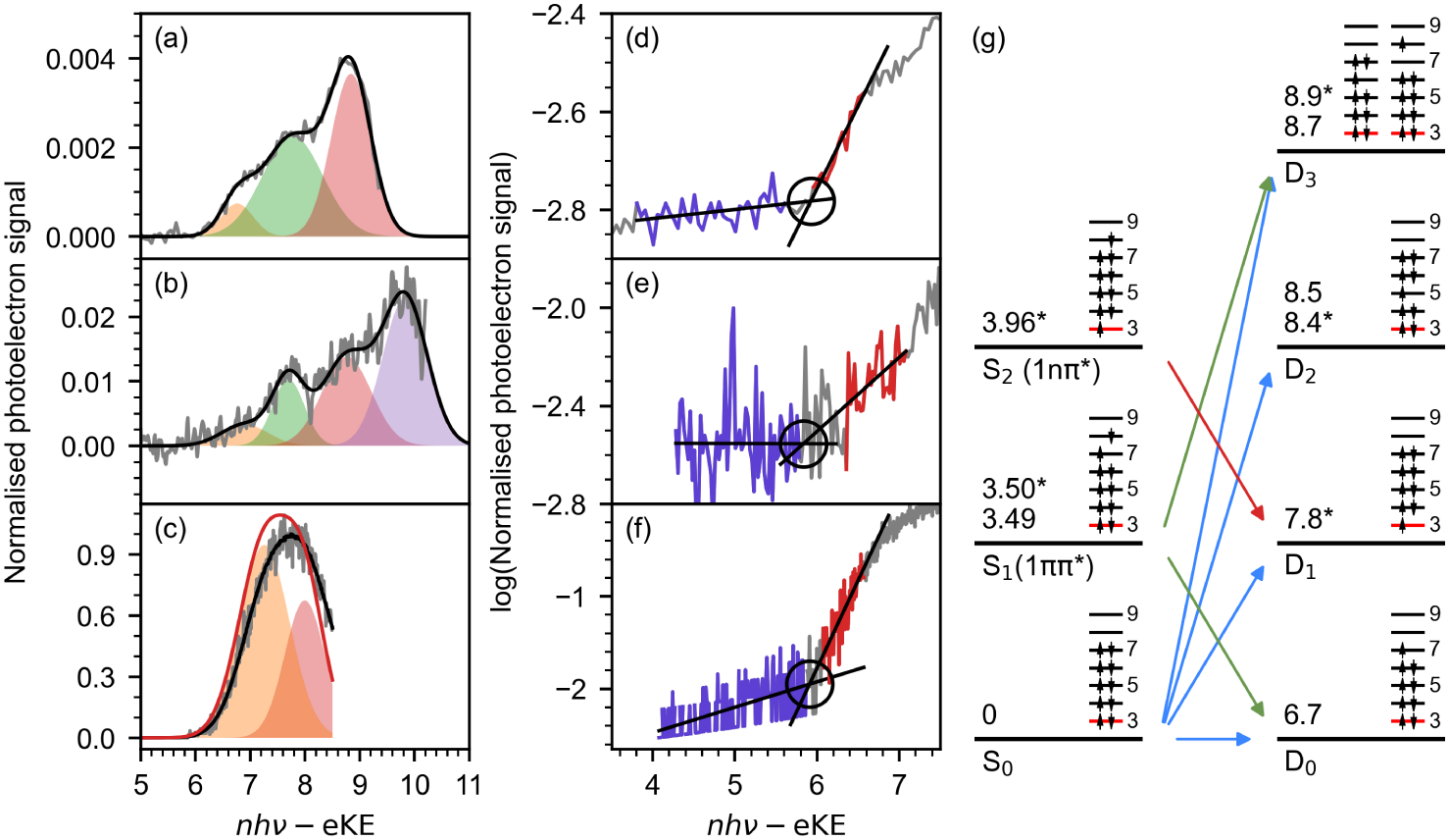
(a, d) X-ray photoelectron spectrum of 5 mM *p*CE^–^ recorded with a photon energy of 180 eV. (b, e) EUV photoelectron spectrum of 5 mM *p*CE^–^ recorded with a photon energy of 40.81 eV. (c, f) Multiphoton UV photoelectron spectrum of 50 μM *p*CE^–^ recorded with a photon energy of 3.02 eV (410 nm). Spectra are normalized to the maximum of the 1b_1_ valence band of liquid water for X-ray and EUV measurements and to the maximum photoelectron signal for UV measurements. (d–f) Straight line fits (black) to the rising edges of the photoelectron signals (red) and noise baselines (purple) in log space used to estimate adiabatic detachment energies (ADEs). (g) Electronic configurations of the first few electronic states of aqueous *p*CE^–^ and the corresponding neutral radical at the XMCQDPT2/(aug)-cc-pVDZ/EFP level of theory. Vertical excitation energies (VEEs) and VDEs with an asterisk were calculated using a mixed n/π active space, while the rest were calculated using a pure π active space. The horizontal black and red lines labeled with numbers indicate the π and n orbitals respectively, and their occupation is shown for each state. Arrows indicate likely detachment processes based on the electronic configuration of the states involved.

The X-ray difference spectrum of 5 mM *p*CE^–^ was fit with three Gaussians below 9.35 eV (Figure 2a). Above this high eBE cutoff, the difference spectrum is dominated by the noise of the signal. The dependence of the centers of the Gaussians on the value of the high eBE spectral cutoff is shown in Figure S8. We quote the first two VDEs of *p*CE^–^ using the values obtained from the fit of the subtracted data: 6.76 ± 0.13 eV and 7.78 ± 0.13 eV, and note that the lowest VDE is very similar to that of the green fluorescent protein chromophore in aqueous solution.^55^ The position of the red Gaussian is sensitive to the value of the high eBE cutoff and is quoted as 8.8 ± 0.2 eV to reflect this.

The eKEs measured in the EUV spectrum of 5 mM *p*CE^–^ are >15 eV, so the valence signal is essentially free from distortion caused by inelastic electron scattering.^34,35^ The difference spectrum was fit with four Gaussians. An unconstrained fit resulted in the two lowest energy Gaussians having the same eBE (∼7.7 eV) but different fwhm (2 and 0.2 eV). These fwhm are inconsistent with typical values for aqueous solutions of organic solutes. Constraining the fit so that the fwhm of the lowest eBE peak has a maximum of 1 eV resulted in the first three peaks having eBEs of 6.9 ± 0.2, 7.7 ± 0.1, and 8.8 ± 0.1 eV, which are consistent with those of the subtracted X-ray data. A peak at 9.8 eV is present in the EUV spectrum that is not observed in the X-ray spectrum and cannot be assigned. A possible explanation is that there are overlapping contributions from OH^–^ (VDE = 9.2 eV)^56,57^ or higher VDEs of *p*CE^–^.

The central VDEs obtained from the X-ray and EUV spectra are summarized in Table 1, and are in good agreement with those determined at the XMCQDPT2/(aug)-cc-pVDZ/EFP level of theory. We note that when a pure π valence active space is used, the calculated VDEs for the D_2_ (8.5 eV) and D_3_ (8.7 eV) channels differ by only 0.2 eV. Consequently, the corresponding experimental bands are expected to overlap, making a definitive assignment between D_2_ and D_3_ difficult.

**Table 1:** Lower-Energy VDEs Together with Corresponding ADEs of Aqueous *p*CE^–^ Determined Using X-Ray, EUV, and Multiphoton UV LJ-PES, Together with XMCQDPT2/(aug)-cc-pVDZ/EFP Calculated VDEs^a^

		S_0_–D_0_		S_0_–D_1_	S_0_–D_2_/D_3_
Technique	*h*ν	VDE	ADE	VDE	VDE
X-ray LJ-PES	180	6.76 ± 0.13	5.9	7.78 ± 0.13	8.8 ± 0.2
EUV LJ-PES	40.81	6.9 ± 0.2	5.8	7.7 ± 0.1	8.8 ± 0.1
UV LJ-PES	3.02	–	5.9	–	–
Calculated	–	6.7	–	7.8	8.5/8.7

a All values are in eV.

Vertical ionization energies and VDEs can be determined using UV femtosecond laser pulses by recording photoelectron spectra with wavelengths that are nonresonant with excited states.^38,41^ Since *p*CE^–^ has a significant absorption cross section in aqueous solution at wavelengths below 400 nm, multiphoton detachment avoiding this resonance requires three photons. However, *p*CE^–^ also absorbs light at wavelengths just above 200 nm (Figure 1). A consequence of this is that two-photon resonances make nonresonant multiphoton detachment PES impossible for *p*CE^–^. The UV photoelectron spectrum of 50 μM *p*CE^–^ recorded with 410 nm is presented in Figure 2c. It is worth highlighting that although the concentration of *p*CE^–^ is 2 orders of magnitude lower in these measurements than in the X-ray and EUV experiments, the signal-to-noise ratio is excellent due to the absence of a water photoelectron signal—photoionization of water would require four photons at 410 nm—and the comparatively narrow spectral width of the multiphoton detachment spectra. Employing our spectral retrieval software LJscatter^41^ to model the inelastic scattering of electrons with eKE <5 eV, with a concentration depth profile for *p*CE^–^ determined using molecular dynamics simulations (Figure S9), we fit two Gaussians to the spectrum and obtained values for 3*h*ν - eKE of 7.3 ± 0.1 eV and 8.0 ± 0.1 eV. These values are higher than the one-photon VDEs obtained from the X-ray and EUV spectra and are not included in Table 1. However, such resonant multiphoton detachment measurements reveal information about the VDEs from excited electronic states and this is discussed in detail below.

Photoelectron spectra can also be employed to approximate adiabatic detachment energies (ADEs), by determining the eBE corresponding to the onset of the photoelectron signal. A common procedure involves fitting a straight line to the rising edge of the signal and the noise baseline in log space.^58–60^ Figure 2d–f shows this procedure for the X-ray (180 eV), EUV (40.81 eV), and UV (3.02 eV) photoelectron spectra of *p*CE^–^. The signal and noise baselines are highlighted in red and purple, respectively, and the intersections are marked with black circles. The ADEs determined from the three spectra (Table 1) are in agreement with one another (∼5.8 eV). It should be noted that this value is effectively the threshold for electron detachment rather than a “true” ADE. This analysis assumes that, upon photodetachment, changes in solute equilibrium geometry and solvent relaxation are sufficiently small that the Franck–Condon profile extends to the equilibrium geometry of the solvated neutral radical. We also note that hot transitions dominate the onset of the photoelectron band and depend on temperature. The similarity between the UV, X-ray, and EUV measurements suggests that all three spectroscopic methods can be used to estimate ADEs from the ground electronic state.

### Resonant Detachment

To interpret the resonant multiphoton PES of aqueous *p*CE^–^ requires knowledge of its electronic structure. The first four vertical excitation energies (VEEs) and VDEs of *p*CE^–^ in aqueous solution were determined at the XMCQDPT2/(aug)-cc-pVDZ/EFP level of theory (Figure 2g). To a first approximation, we can use Koopmans’ theorem to determine propensities for one-photon direct detachment from various electronic states of the anion. For example, from the electronic configurations shown in Figure 2, we see that the S_0_ state is likely to ionize to D_0_, D_1_, D_2_, and D_3_, S_1_ to D_0_ and D_3_, and S_2_ to D_1_.

We now turn to the multiphoton UV PES measurements. Figure 3 shows multiphoton photoelectron spectra of *p*CE^–^ recorded with wavelengths between 410 nm (3.02 eV) and 310 nm (4.00 eV) to access the first two electronically excited states (Figure 1) that ionize to D_0_ (VDE ∼6.7 eV), D_1_ (VDE ∼7.8 eV) and D_3_ (VDE ∼8.8 eV) (Figure 2g). The spectra were fit with distorted Gaussians using our spectral retrieval software LJscatter.^41^ The 400 nm spectrum was fit with two Gaussians; the 380, 365, and 350 nm spectra were fit with three Gaussians; and the 310 nm spectrum was fit with one Gaussian. Although the 310 nm spectrum also has a small contribution on the high eKE side of the main peak (magnified 20 times in Figure 3e), which corresponds qualitatively to the red and orange peaks present in the other spectra, the low intensity of these features in this spectrum makes fitting them unreliable. Whereas the higher energy red and orange peaks are present in all spectra, the low energy blue peak is not observed at all in the 410 and 400 nm spectra, it is first observed in the 380 nm spectrum and its contribution increases as the wavelength decreases until it dominates the spectrum at 310 nm (4 eV). This suggests that it arises from a near-threshold detachment process.

**Figure 3:**
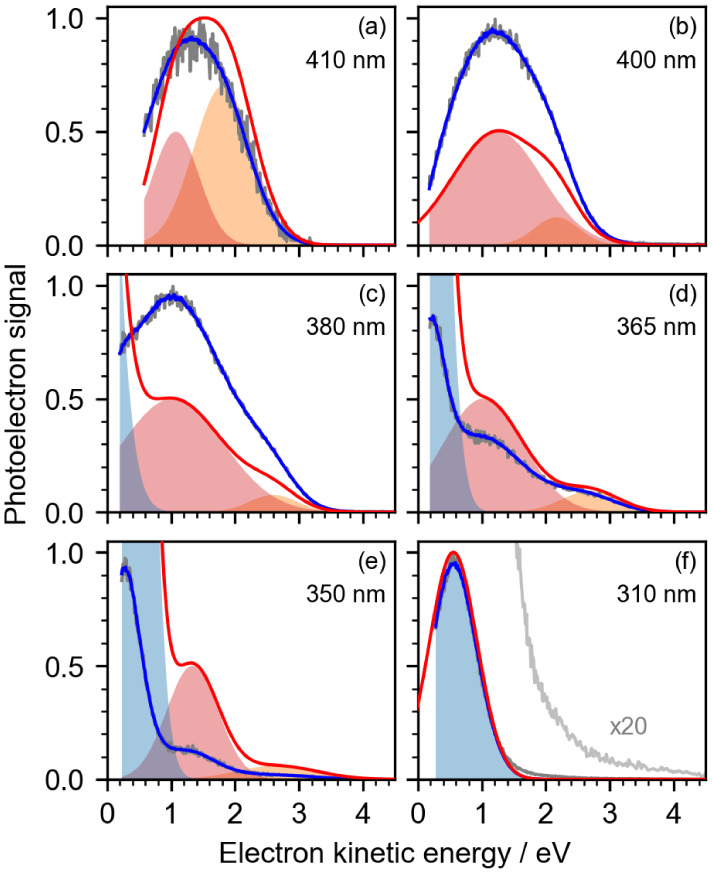
Photoelectron spectra of *p*CE^–^ recorded at stated wavelengths (gray). The (a) 410 nm and (b) 400 nm spectra of 50 μM *p*CE^–^ in 3.5 mM aqueous NaOH were fit with two Gaussians using LJscatter. The (c) 380 nm, (d) 365 nm, and (e) 350 nm spectra of 50 μM *p*CE in 1.0 mM aqueous NaOH were fit with three Gaussians. The (f) 310 nm spectrum of a 50 μM *p*CE in 2.0 mM aqueous NaOH was fit with one Gaussian. Blue lines are the fits to the data and red lines are the retrieved spectra.

To investigate the nature of the detachment processes observed when using resonant wavelengths, the dependence of the photoelectron spectrum of *p*CE^–^ on the power and wavelength of the laser pulses was investigated. Figure 4a is a log–log plot of peak area as a function of laser pulse energy for the three features present in the 350 nm photoelectron spectrum of *p*CE^–^. The peak areas were determined from fits of three Gaussian eKE distributions with peak centers and fwhm fixed to be the same as those obtained from the 350 nm photoelectron spectrum of *p*CE^–^ in Figure 3. Slopes of 1.61 ± 0.06, 2.71 ± 0.09, and 2.61 ± 0.07 were obtained by fitting straight lines to the data in Figure 4a, suggesting that the low eKE peak arises from a two-photon process and the two higher eKE peaks arise from three-photon processes. The deviation from integer values is attributed to the fact that photodetachment occurs via resonances and there are different transition probabilities for absorption and detachment.^61^ This is consistent with our observations (Figure 3): the 400 nm spectrum is dominated by resonance-enhanced detachment via S_1_ with three photons because resonance-enhanced detachment via S_1_ with two photons is not possible energetically, then as the wavelength decreases, the competing two-photon process begins to appear at very low eKE and its relative intensity increases with decreasing wavelength until it eventually dominates the spectrum recorded at 310 nm.

**Figure 4:**
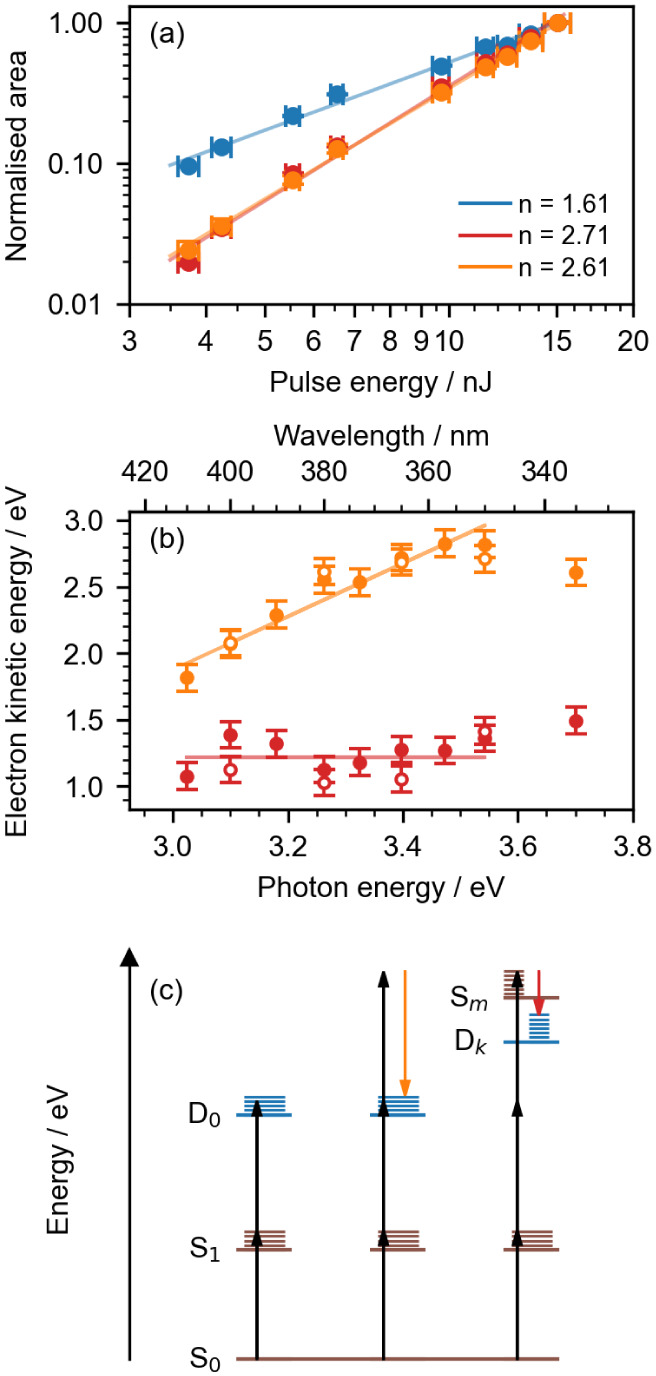
(a) Log–log plot of the peak area of the retrieved Gaussians normalized to the area at highest pulse energy, as a function of pulse energy for the three peaks in the 350 nm spectrum of *p*CE^–^. Blue, red, and orange circles represent the area of the low, middle, and high eKE peaks. Solid lines are the linear fits. (b) Central eKE of the high (orange) and middle (red) eKE peaks of the photoelectron spectra of *p*CE^–^ between 410 and 335 nm as a function of photon energy. Full and empty circles are data obtained from the fits of spectra recorded on two different days. The red and orange straight lines have slopes of 0 and 2, respectively; note that the orange line is not a fit to the data. (c) Scheme highlighting the processes that give rise to the low (blue), middle (red), and high (orange) eKE peaks. S*_m_* is an excited state of *p*CE^–^ embedded in the detachment continuum and D*_k_* is an electronically excited state of the neutral radical of *p*CE^–^.

As the two higher eKE peaks arise from three-photon resonance-enhanced detachment processes, plotting their central eKE as a function of photon energy can provide valuable information about the two-photon detachment process. Figure 4b is a plot of central eKE as a function of photon energy in which the peak centers from Figure 3 are shown as empty circles and the peak centers obtained from the 410 nm spectrum in Figure 2 and from additional measurements (Figure S10) are shown as full circles. The central eKE of the red peak remains approximately constant for photon energies below ∼3.6 eV, whereas the central eKE of the orange peak increases at twice the rate of the increase in photon energy.

The constant eKE of the red peak as a function of photon energy is a signature of a three-photon resonance embedded in the detachment continuum, from which indirect detachment occurs. Its value (eKE ≈1.2 eV) represents the energy gap between the three-photon resonance S_*m*_, and the resulting neutral radical D_*k*_ (Figure 4c). This behavior can be rationalized in terms of Franck–Condon factors governing the transition from S_*m*_ to D_*k*_. If the equilibrium geometry shift between S_*m*_ and D_*k*_ is small along all normal modes, as is expected for biological chromophores upon electron detachment from a shape resonance, then the 0–0 transition remains the most intense. Furthermore, if higher vibrational levels are not significantly populated upon excitation to S_*m*_, the 0–0, 1–1, and 2–2 transitions from S_*m*_ to D_*k*_ all have comparable maximum intensities, yielding the same electron kinetic energy regardless of the excitation energy. A detailed discussion of this Franck–Condon analysis can be found in our previous work.^62^

In the gas phase, molecular anions that do not experience large geometry changes upon photodetachment have a propensity to conserve vibrational energy.^63^ This behavior was observed for isolated *p*CE^–^ using anion photoelectron spectroscopy,^16^ and will also be observed for *p*CE^–^ in solution if solvent relaxation is slower than the effective duration of the multiphotion detachment process (∼300 fs). The trend of the eKE of the orange peaks supports this (Figure 4b): the vibrational energy gained upon excitation to S_1_ is conserved upon detachment and the measured eBE of S_1_ (2*h*ν - eKE) is independent of photon energy (Figure S12), with an average value of 4.1 ± 0.1 eV. The corresponding 3*h*ν-eKE values range from 7.2 and 7.8 eV (Figure S12). Although the UV photon energy is less than the vertical eBE of S_1_, it is greater than the threshold for detachment so the process giving rise to the orange peaks can be considered an above threshold ionization process.

In the absence of resonances at the two-photon level, the average eBE of S_1_ would represent the difference between the D_0_ VDE and the adiabatic excitation energy (AEE) of S_1_:

1 VDE−AEE≈2hν−eKE

The AEE is approximately the average of the absorption and emission maxima (Figures 1 and S9). Substituting AEE ∼3.1 and 2*h*ν - eKE = 4.1 eV in eq 1 gives VDE ∼7.2 eV, which is 0.4 eV higher than the value determined from X-ray measurements. This difference can be explained in terms of different overlaps with D_0_ in two-photon detachment from S_1_ and one-photon detachment direct from S_0_. The increase in VDE for two-photon detachment from S_1_ is consistent with an increase in the fwhm of the orange peaks in the UV PES, which range from 0.8 to 1.5 eV for the 400–350 nm spectra, compared to the fwhm of the lowest energy peak in the X-ray spectrum, which is 0.7 eV.

We now turn to the blue peak in the UV photoelectron spectra of *p*CE^–^. Due to the eKE of the blue peak being ∼0 eV in most spectra, an accurate eKE value can only be obtained from the 310 nm data (Figure 3e), giving *h*ν – eKE = 3.4 ± 0.1 eV. The photoelectron distribution of a two-color photoelectron spectrum recorded following excitation to S_1_ with 350 nm and detachment with 266.7 nm (Figure S11) gives *h*ν – eKE = 3.7 ± 0.1 eV, which is 0.3 eV higher than the value determined from the 310 nm spectrum. We attribute this difference to competing detachment processes from S_1_ and S_2_. The S_2_ absorption band can be seen as a small shoulder around 310 nm in Figure 1 and does not have a counterpart in the fluorescence spectrum (Figure S13). The S_2_ state has nπ * character and although the calculated oscillator strength of the S_0_-S_2_ transition is near-zero,^15,17,64^ it can gain intensity by vibronic coupling. Experimental evidence for population of the nπ * state include a smaller solvatochromic shift of the absorption band in ethylene glycol and dimethylformamide solutions compared to water, and the fluorescence anisotropy decreasing rapidly at excitation wavelengths shorter than around 320 nm.^28^ Based on Koopmans’ correlations (Figure 2g), detachment from S_2_ is expected to form the neutral radical in its first excited state (D_1_). The decrease in *h*ν – eKE between the 350 and 310 nm spectra could then be attributed to overlap between signals from these two channels, and the S_2_-D_1_ VDE being lower than the S_1_-D_0_ VDE. Moreover, for one-photon detachment, eq 1 becomes VDE – AEE ≈ *h*ν – eKE, and gives VDE ≈ 6.8 eV, which is the same as the value determined from our X-ray measurements.

The positions of the red and orange peaks in the 335 nm spectrum (Figure 4), which correspond to detachment from S_1_, deviate from the trend observed at wavelengths longer than 350 nm. Furthermore, the one-photon eBE of the blue peak obtained from the two-color spectrum after excitation at 350 nm (3.7 ± 0.1 eV) is about 0.4 eV smaller than the average two-photon eBE of the orange peak (4.1 ± 0.1 eV), despite both originating from S_1_-D_0_ detachment. It is possible that the deviation from the trend is due to a change between two-photon S_1_-D_0_ detachment above 335 nm to one-photon S_1_-D_0_ detachment at 335 nm because *h*ν + AEE = 6.8 eV, which is just enough to cause one-photon detachment out of S_1_. However, transient absorption experiments of *p*CE^–^ and PYP in solution^10,29^ have reported the formation of solvated electrons as a consequence of the absorption of two pump photons. This process was identified by the quadratic dependence of the solvated electron signal on the pulse energy of the exciting laser. Since both *p*CE^–^ and PYP have an excited-state absorption near the S_0_-S_1_ absorption maximum,^10,29,30^ transient absorption measurements led to the suggestion that solvated electrons originate from a high-lying state near the detachment continuum. In our PES measurements, excitation to this state could result in a different overlap with D_0_, thereby influencing the amount of vibrational energy conserved upon detachment and the observed eKE. Consequently, the three-photon detachment to D_0_ could proceed via a sequential mechanism: initial excitation to S_1_, followed by further excitation to a high-lying state near the detachment continuum and, finally, detachment to D_0_.

## Conclusion

The first two vertical detachment energies of the model photoactive yellow protein chromophore *p*CE^–^ have been determined using X-ray and EUV liquid-microjet photoelectron spectroscopy. VDEs of 6.8 ± 0.1 eV and 7.8 ± 0.1 eV were obtained and these are in excellent agreement with our XMCQDPT2/(aug)-cc-pVDZ/EFP calculated values (6.7 and 7.8 eV). The onsets of the X-ray, EUV, and UV photoelectron spectra were also employed to estimate the first ADE of *p*CE^–^ (∼5.9 eV).

The photodetachment pathways of *p*CE^–^ following excitation to its first excited state were investigated with UV LJ-PES across a wavelength range of 410–310 nm. Power-dependent measurements were employed to distinguish between one and two-photon detachment channels and wavelength-dependent measurements disentangled the role of two distinct two-photon detachment mechanisms from S_1_. The first involves direct detachment via a high-lying excited state near the detachment continuum, previously implicated in the formation of solvated electrons. The presence of this high-lying resonance was inferred from the difference between the one-photon and two-photon eBEs of S_1_ and the change in slope of the eKE of the two-photon channel as a function of photon energy. The second two-photon process involves autodetachment from a high-lying excited state embedded within the detachment continuum. Such resonances are often observed in isolated anions in the gas phase which have low detachment energies, and this work provides experimental evidence for the existence of similar low-lying resonances just above the detachment threshold of anions in aqueous solution.

## Supplementary Material



## Notes

Measurement of vacuum level offset; NMR spectra; computational details; procedures for fitting X-ray photoelectron spectra; molecular dynamics simulations of solute depth profile; additional multiphoton UV photoelectron spectra; eBE trends; and fluorescence spectrum (PDF)
