# Supplementary material for: Liquid-Microjet Photoelectron Spectroscopy of the Photoactive Yellow Protein Chromophore in Aqueous Solution
Source: J Phys Chem A. 2026 Jun 12;130(25):4814–22. doi: 10.1021/acs.jpca.6c02627 (PMC13312452; doi:10.1021/acs.jpca.6c02627)
Supplement: Supplementary file 1 [file jp6c02627_si_001.pdf]

Supporting Information: Liquid-microjet Photoelectron Spectroscopy of the Photoactive Yellow Protein Chromophore in Aqueous Solution

Edoardo Simonetti,[†] Anton N. Boichenko,[‡] Johanna Rademacher,[†] Alice Henley,[†] Kate Robertson,[†] Harmanjot Kaur,[¶] Sebastian Malerz,[¶] Iain Wilkinson,[§] Bernd Winter,[¶] Anastasia V. Bochenkova,[‡] and Helen H. Fielding*,[†]

†*Department of Chemistry, University College London, 20 Gordon Street, London WC1H 0AJ, U.K.*

‡*Department of Chemistry, Lomonosov Moscow State University, 119991 Moscow, Russia*

¶*Molecular Physics Department, Fritz-Haber-Institut der Max-Planck-Gesellschaft, 14195 Berlin, Germany*

§*Institute for Electronic Structure Dynamics, Helmholtz-Zentrum Berlin für Materialien und Energie, 14109 Berlin, Germany*

E-mail: h.h.fielding@ucl.ac.uk

Contents

S1 Experimental Details **S1**

S1.1 Measurement of Vacuum Level Offset S1

S1.2 NMR spectra S3

S2 Computational Details **S6**

S3 X-ray Photoelectron Spectra **S7**

S3.1 Full spectrum fits with literature constrained water peaks S7

S3.2 Full and subtracted spectrum fits with free water peaks S8

S3.3 Effect of cutoff on the subtracted spectrum fits S10

S4 Molecular Dynamics Simulations **S11**

S5 Additional Multiphoton UV Photoelectron Spectra **S13**

S6 2$h\nu$ and 3$h\nu$ Electron Binding Energies **S14**

S7 Fluorescence Spectrum **S14**

References **S15**

S1. Experimental Details

S1.1. Measurement of Vacuum Level Offset

When the liquid jet is introduced in the interaction chamber, evaporation of water molecules from its surface results in adsorption of water molecules onto the walls of the chamber which shifts the vacuum level in the interaction chamber. We refer to the difference in vacuum level between the interaction region and the detector as the vacuum level offset V_0. To measure V_0 and the difference between the vacuum level at the surface of the jet and at the skimmer induced by the streaming potential ϕ_{str}, photoelectron spectra of Xe are recorded while the liquid jet is translated set distances from the interaction region along the skimmer magnet axis (Figure S1).

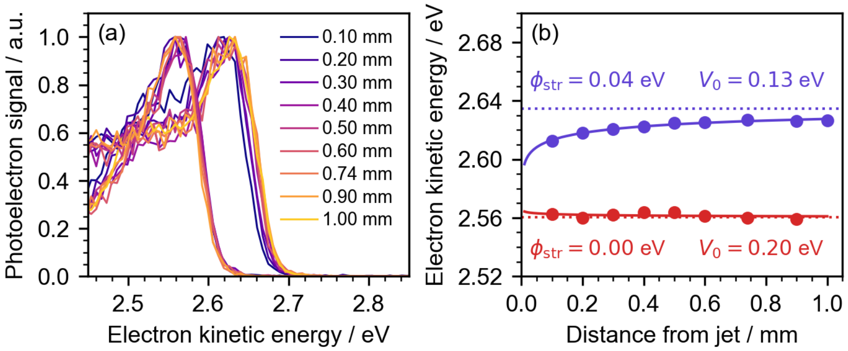

Figure S1: (a) Photoelectron spectra of the $^2\mathrm{P}_{3/2}$ peak of Xe following [2+1] photoionisation at 249.7 nm recorded at set distances between the ionisation point and the liquid jet. The spectra at higher eKEs were recorded before a set of liquid jet measurements and the spectra at lower eKEs were recorded after a set of liquid jet measurements. (b) Measured eKE of the $^2\mathrm{P}_{3/2}$ peak of Xe following [2+1] photoionisation at 249.7 nm as a function of distance from the liquid jet. The data were measured before (violet) and after (red) a set of liquid jet measurements. Solid lines indicate the fit of Equation S1 and dotted lines the asymptote at large distances, which corresponds to $\mathrm{eKE}_{\mathrm{true}} - V_0$.

The rising edge of the Xe $^1\mathrm{S}_0 \rightarrow {}^2\mathrm{P}_{3/2}$ transition are fit with a Gaussian and the peak centres are fit with the following equation[S1]

$$\mathrm{eKE}_{\mathrm{obs}}(x) = \mathrm{eKE}_{\mathrm{true}} - \phi_{\mathrm{str}} \frac{\ln\left(\dfrac{2L + x}{x}\right)}{\ln\left(\dfrac{2(L + x) - r}{r}\right)} - V_0, \tag{S1}$$

where $\mathrm{eKE}_{\mathrm{obs}}(x)$ is the peak eKE when the jet is translated by a distance x away from the interaction region, $\mathrm{eKE}_{\mathrm{true}}$ is the field-free eKE, L is the distance between the ionisation point and the skimmer and r is the radius of the jet. Under experimental conditions, $x = r$, i.e. the potential at the interaction region is given by the potential at the surface of the jet, and Equation S1 reduces to

$$\mathrm{eKE}_{\mathrm{obs}} = \mathrm{eKE}_{\mathrm{true}} - \phi_{\mathrm{str}} - V_0. \tag{S2}$$

Since the vacuum level offset changes during the duration of the measurement as more water molecules adsorb onto the surfaces of the interaction region, the potential in the interaction cannot be fully flattened. Instead, ϕ_{str} is kept below an absolute value of 0.08 eV and it is measured together with the vacuum level offset before and after a set of measurements. An example measurement is shown in Figure S1. Measured photoelectron spectra are shifted by the average of the sums of ϕ_{str} and V_0. The sums typically vary by a few tens of meV over the course of a measurement.

S1.2. NMR spectra

Nuclear magnetic resonance (NMR) spectra of 5.0 mM *p*CE with 5.5 mM NaOD in D_2O, and 50 µM *p*CE with 1.25 mM NaOD in D_2O, are presented in Figures S2 and S3. Neither spectrum shows evidence of aggregation. The NMR spectrum of 5.0 mM $p\mathrm{CE}^-$ has a few extra peaks not present in the spectrum of 50 µM $p\mathrm{CE}^-$ that are consistent with the dianion of para-coumaric acid and methanol, formed by hydrolysis of $p\mathrm{CE}^-$. The ratio of the NMR signals of the methyl groups of methanol and $p\mathrm{CE}^-$ is 0.16:1, and that of the high-ppm vinyl and aromatic protons of the dianion of *p*CA ($p\mathrm{CA}^{2-}$) and $p\mathrm{CE}^-$ is 0.29:1, illustrating that the solution is predominantly that of aqueous $p\mathrm{CE}^-$. This analysis highlights the advantage of being able to use significantly lower concentrations to minimise aggregation and competing chemical reactions.

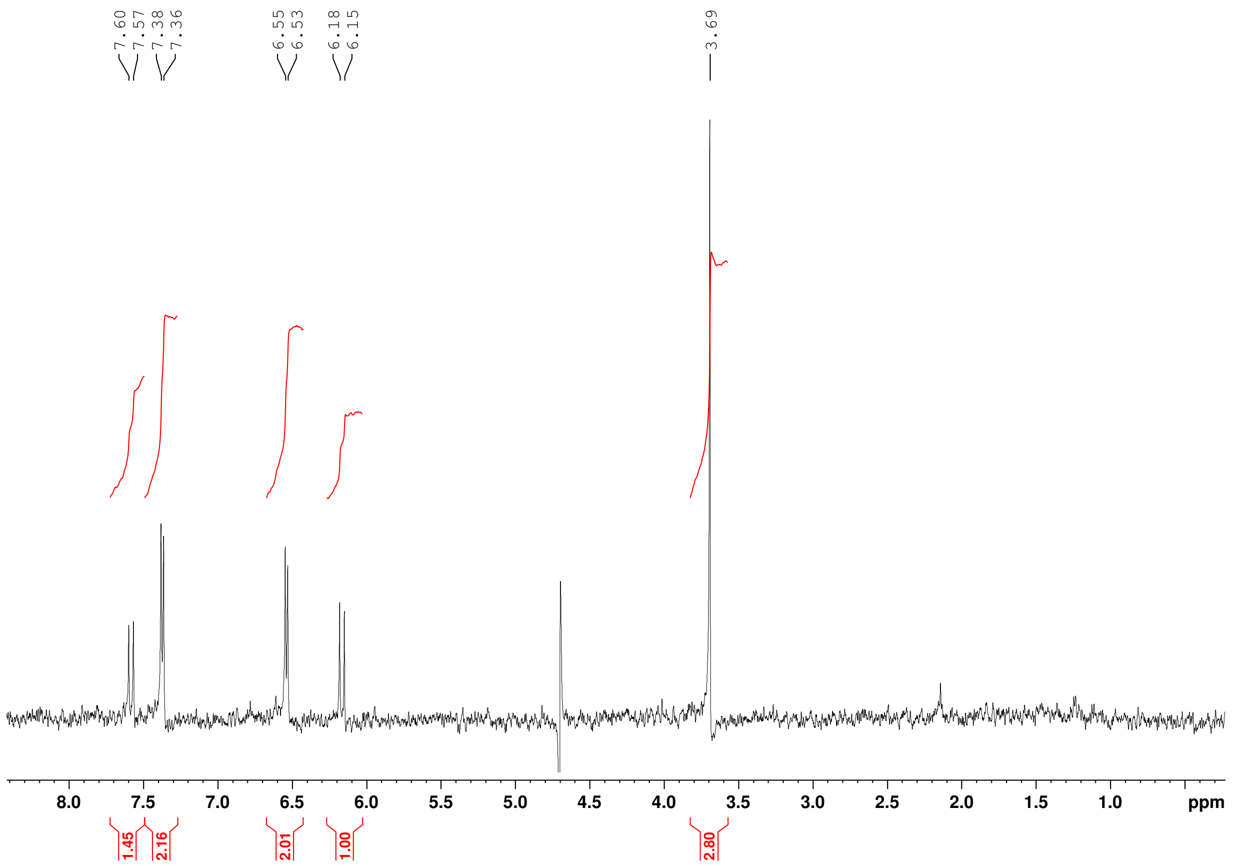

Figure S2: ^{1}H NMR spectrum (500 MHz, D_2O) of 50 µM *p*CE in 1.25 mM NaOD. The contribution from H_2O, HDO and OD^- has been subtracted.

^{1}**H NMR** (500 MHz, D_2O) of 50 µM *p*CE in 1.25 mM NaOD: δ 7.58 (d, J = 15.8 Hz, 1H), 7.37 (d, J = 8.6 Hz, 2H), 6.54 (d, J = 8.5 Hz, 2H), 6.16 (d, J = 15.8 Hz, 1H), 3.69 (s, 3H).

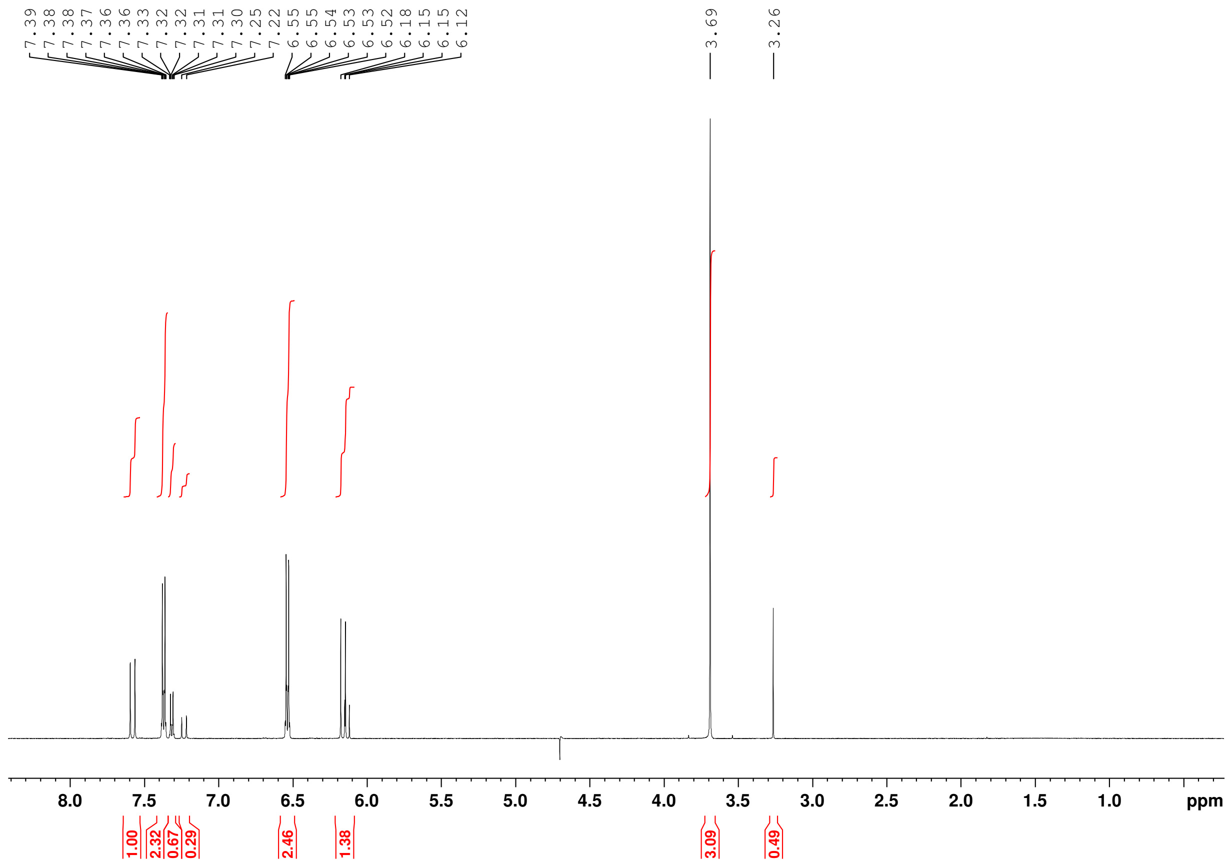

Figure S3: ^{1}H NMR spectrum (500 MHz, D_2O) of 5 mM *p*CE in 5.5 mM NaOD. The contribution from H_2O, HDO and OD^- has been subtracted.

^{1}**H NMR** (500 MHz, D_2O) of 5 mM *p*CE in 5.5 mM NaOD: *p*CE^- δ 7.58 (d, J = 15.9 Hz, 1H), 7.37 (ddd, J = 8.7 Hz, 2H), 6.54 (d, J = 15.5 Hz, 2H), 6.16 (d, J = 15.9 Hz, 1H), 3.69 (s, 3H); *p*CA^{2-} δ 7.32 (ddd, J = 8.7 Hz, 2H), 7.23 (d, J = 15.9 Hz, 1H), 6.14 (d, J = 15.9 Hz, 1H), 3.26 (s, 3H); MeOH δ 3.26 (s, 3H).

S2. Computational Details

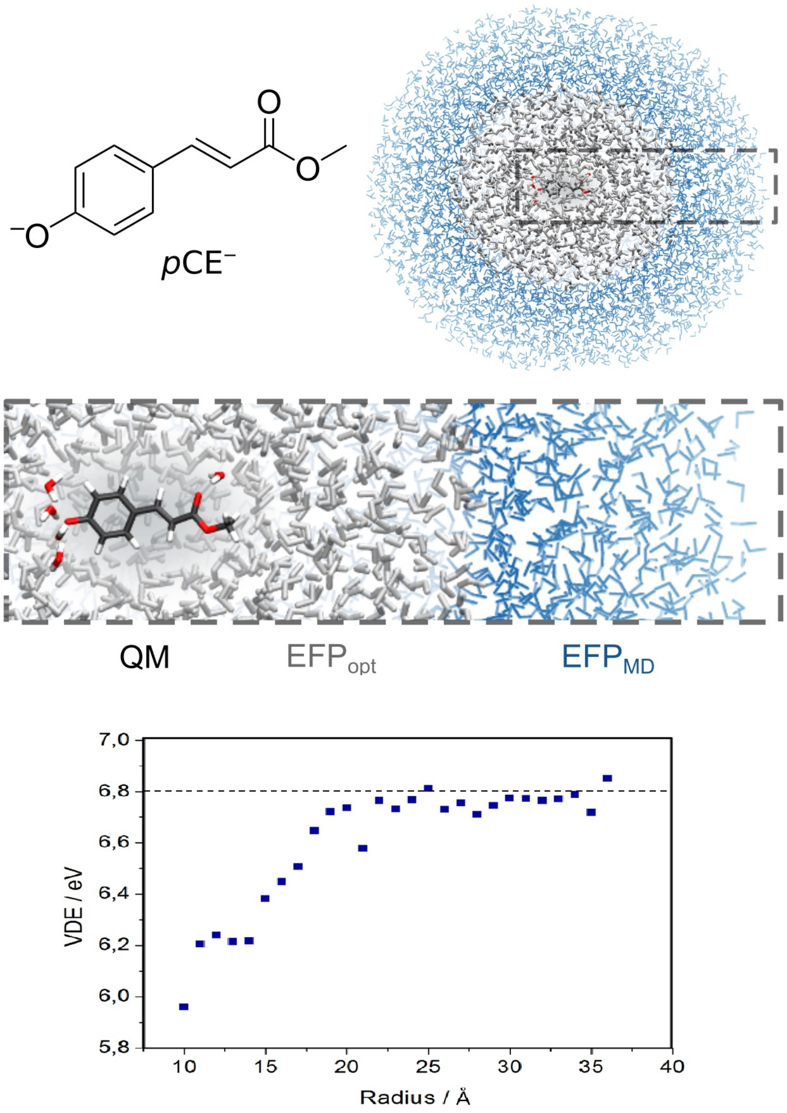

Figure S4: Top left: structure of model PYP chromophore pCE^{-}. Top right: model solvated system with an optimised QM(pCE$^{-}\cdot(H_2O)_5$)/EFP$_{\text{opt}}$ ($\sim$ 1000 H_2O) core surrounded by further water molecules with positions only relaxed within a classical force field, EFP$_{\text{MD}}$. The dashed rectangular box shows the zoomed-in region. Bottom: size convergence as the system is extended from just the optimised core out to R = 36 Å ($\sim$ 10600 H_2O). Horizontal dashed black line indicates the converged VDE of 6.8 eV. A structure with a total of ~7,700 water molecules (R = 34 Å), which showed the converged D_0 VDE at the PBE0/(aug)-cc-pVDZ level of theory, was then used for higher-level XMCQDPT2/EFP calculations of the first and higher-lying VDEs. The details of the computational protocol used to construct the solvated model can be found in Ref. S2.

S3. X-ray Photoelectron Spectra

S3.1. Full spectrum fits with literature constrained water peaks

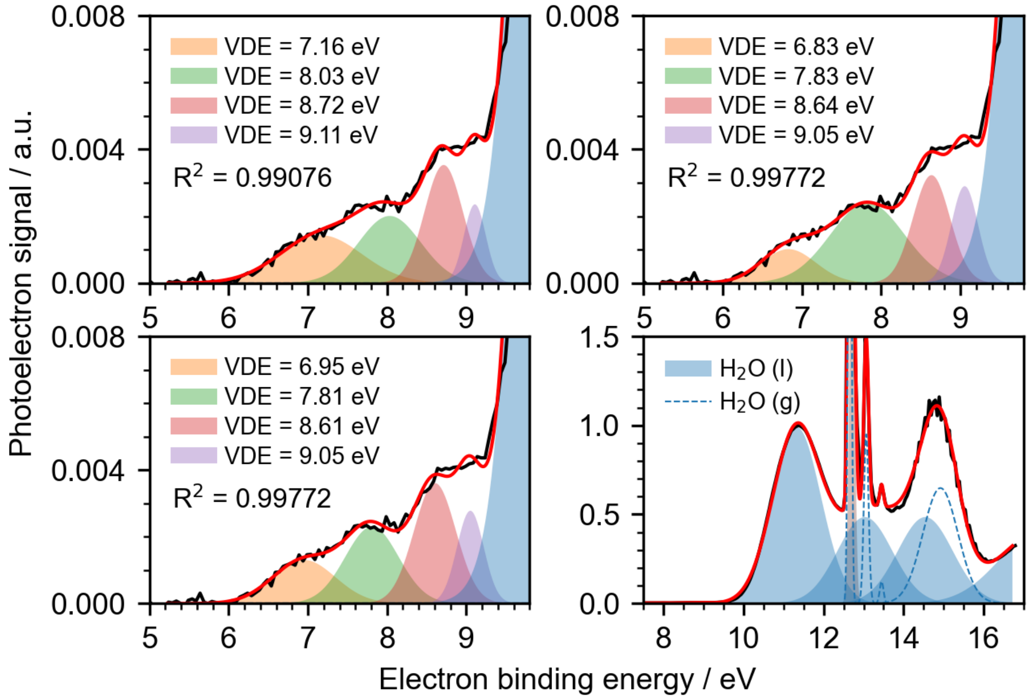

Figure S5: Possible fits of the X-ray ($h\nu$ = 180 eV) photoelectron spectrum of 5 mM *p*CE in aqueous solution with 5.5 mM NaOH and 30 mM NaF, in which the widths and relative positions of the liquid water Gaussians were constrained to literature values.[S3,S4] The parameters of the gaseous water and *p*CE$^-$ Gaussians were not constrained. The eKE was calibrated to the $1b_1$ peak of water, which was set to 11.33 eV.[S3] The fits are sensitive to the initial guess and several sets of parameters describe the contribution of *p*CE$^-$ adequately. The parameters describing liquid and gaseous water are virtually unaffected.

S3.2. Full and subtracted spectrum fits with free water peaks

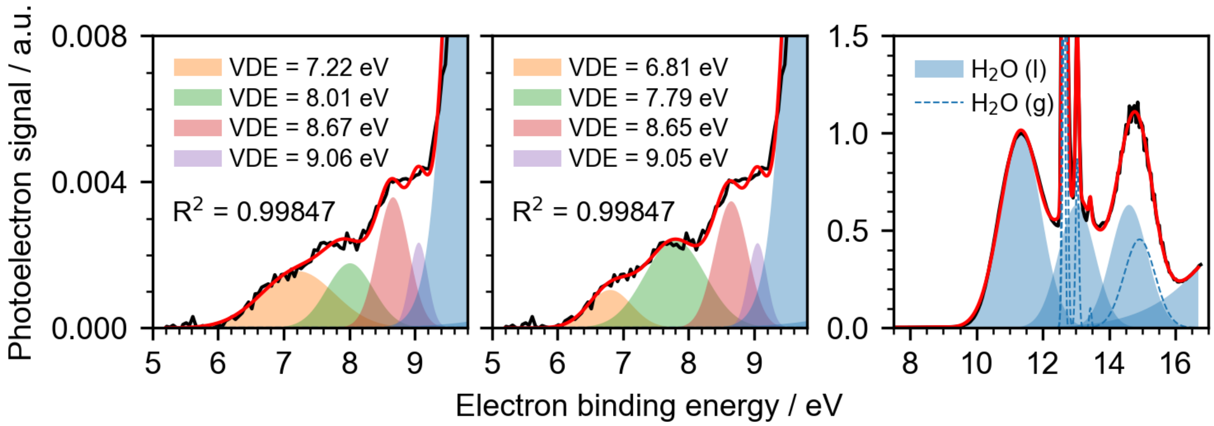

Figure S6: Possible fits of the X-ray ($h\nu$ = 180 eV) photoelectron spectrum of 5 mM pCE in aqueous solution with 5.5 mM NaOH and 30 mM NaF, in which the only widths and relative intensities of the $3a_1$ liquid water Gaussians were constrained to be the same.[S3] The parameters of the other peaks were free. The eKE was calibrated to the $1b_1$ peak of water which was set to 11.33 eV.[S3] The fits are sensitive to the initial guess and several sets of parameters describe the contribution of pCE$^-$ adequately. The parameters describing liquid and gaseous water are virtually unaffected.

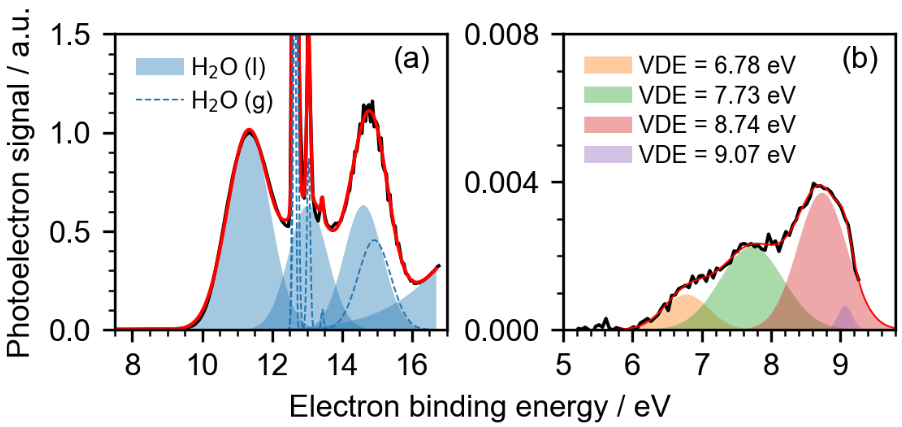

Figure S7: X-ray photoelectron spectrum of 5 mM *p*CE in aqueous solution with 5.5 mM NaOH and 30 mM NaF recorded with a photon energy of 180 eV. (a) The spectrum was fit as a sum of Gaussians representing photoionisation of liquid and gaseous water and photodetachment of *p*CE^- and a vertical offset to capture the baseline noise. The widths and relative intensities of the $3a_1$ liquid water Gaussians were constrained to be the same.[S3] The parameters of the other peaks were free. The eKE was calibrated to the $1b_1$ peak of water which was set to 11.33 eV.[S3] (b) The water contribution to the spectrum was subtracted and the signal below 9.35 eV (black line) was fit with four Gaussians. The obtained VDEs of *p*CE^- are in excellent agreement with those obtained from constraining the liquid water Gaussians to the literature values (Figure 2 in the main text).

S3.3. Effect of cutoff on the subtracted spectrum fits

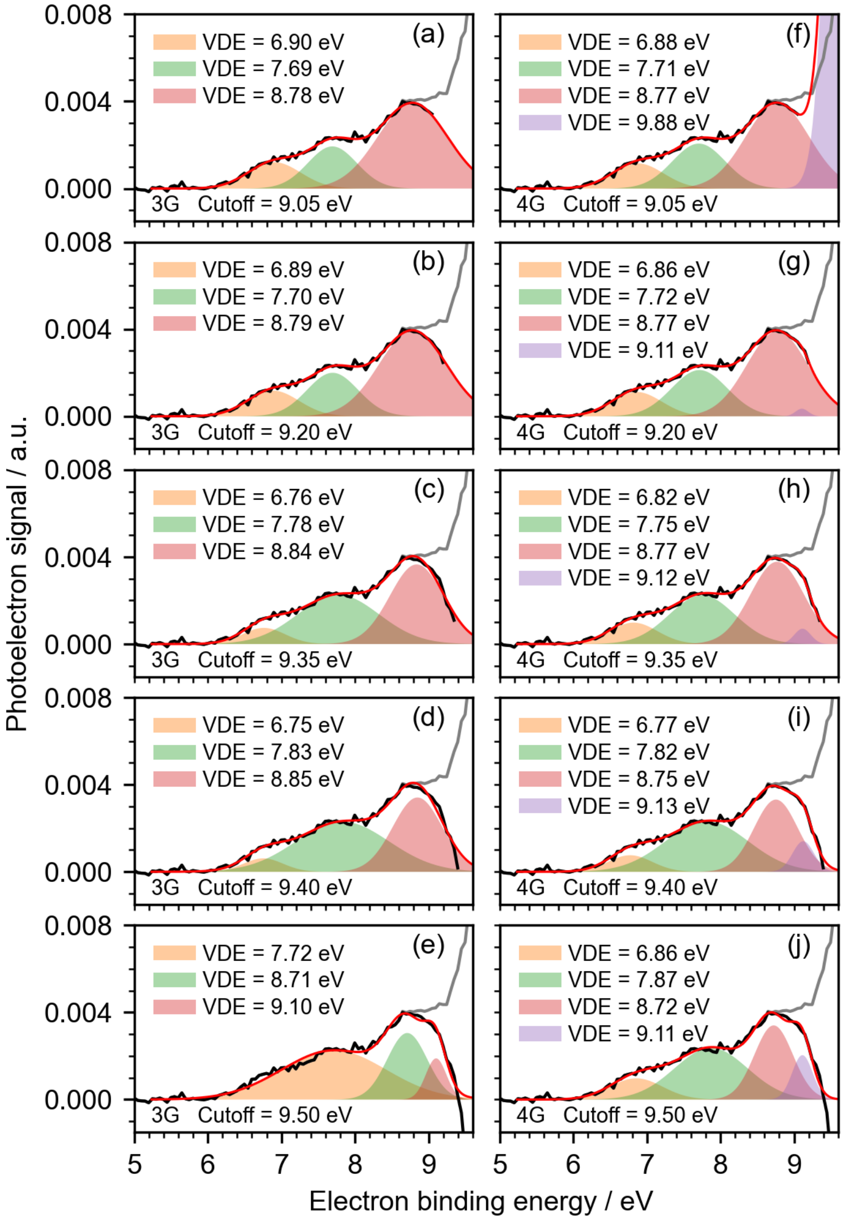

Figure S8: Effect of the high eBE cutoff on the fits of the X-ray ($h\nu = 180$ eV) photoelectron spectrum of 5 mM *p*CE in aqueous solution with 5.5 mM NaOH and 30 mM NaF from which the water contribution was subtracted. Left: three Gaussians were employed. Gray lines represent the measured spectrum, black lines represent the subtracted spectrum below the cutoff, and red lines represent the fits. Right: four Gaussians were employed. Each row corresponds to a different cutoff value, shown in increasing order from top to bottom: 9.05, 9.20, 9.35, 9.40, and 9.50 eV.

S4. Molecular Dynamics Simulations

Simulations used the OpenMM package (version 7.7.0).[S5] Initial coordinates of $p\mathrm{CE}^-$ were optimised using the Automated Topology Builder and Repository[S6,S7] at the B3LYP/6-31G* level of theory. The SMIRNOFF force field (version 0.11.4)[S8] was employed to model the solute surrounded by a Transferable Intermolecular Potential with 3 Points (TIP3P) water environment.[S9] A $p\mathrm{CE}^-$ molecule and Na^+ ion were placed in a $5 \times 5 \times 9$ nm box and surrounded with water molecules. The box was then expanded to $5 \times 5 \times 18$ nm to simulate the liquid-vacuum interface and periodic boundary conditions were applied. This results in an infinite series of slabs with a thickness of 9 nm, separated by 9 nm of vacuum. MD simulations were run using Langevin dynamics with LFMiddle discretisation[S10] with a heat bath set at 300 K, a 2 fs time step and a friction coefficient of 1 ps^{-1}. Long-range electrostatic interactions were described by the Particle Mesh Ewald (PME) with a 1 nm cutoff. A local energy minimization was performed and the velocities of all the atoms were set to random values chosen from the Boltzmann distribution at 300 K, before the system was allowed to equilibrate for 2 ns. Simulations were run for 120 ns, recording the position of all atoms every 4 ps. Six simulations were carried out. The centre of mass of the solute was used to track its position along the axis perpendicular to the surface.

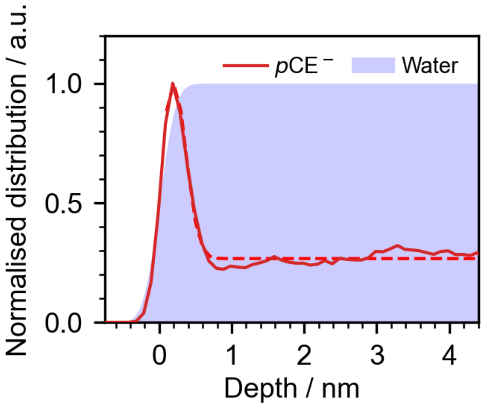

Figure S9: Normalised concentration depth profile of $p\mathrm{CE}^{-}$ in aqueous solution at the liquid-vacuum interface. The blue shaded area is the cumulative normal distribution fit to the distribution of the oxygen atoms of water molecules and normalised to 1. The dashed line indicates the concentration depth profile employed in the retrieval of $p\mathrm{CE}^{-}$ spectra.

S5. Additional Multiphoton UV Photoelectron Spectra

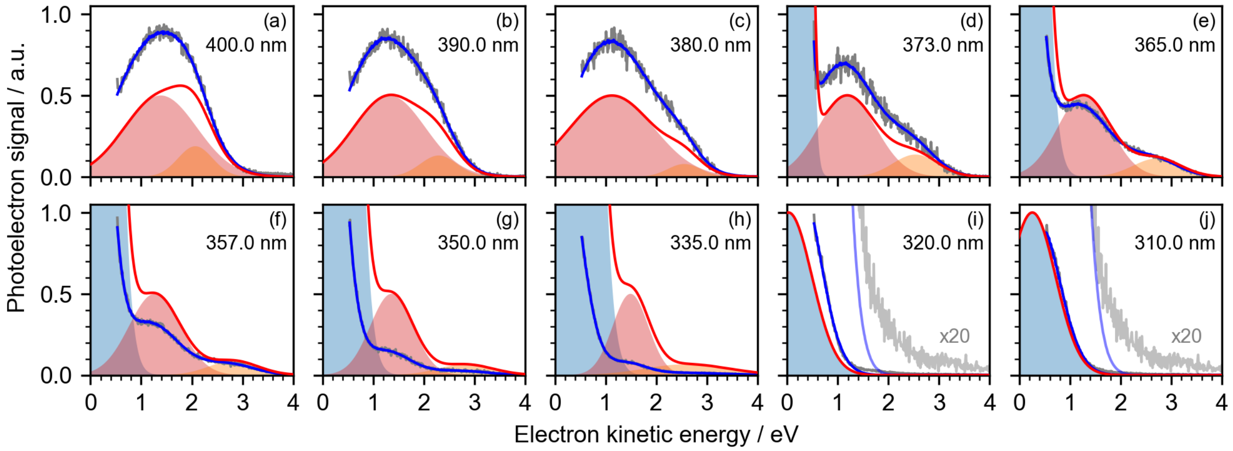

Figure S10: Photoelectron spectra of a 50 µM *p*CE 1.0 mM NaOH solution recorded with specified wavelengths (gray). The spectra in (a-c) were fit with two Gaussians. The spectra in (c-h) were fit with three Gaussians. The spectra in (i, j) were fit with one Gaussian. Blue lines are the fit to the data and red lines are the retrieved spectra.

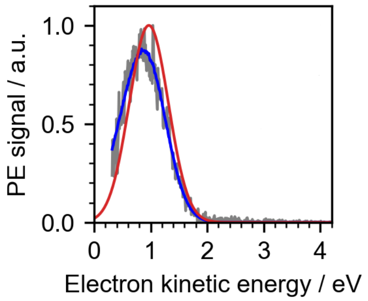

Figure S11: (a) Two-colour photoelectron spectrum of aqueous $p\text{CE}^-$ recorded following photoexcitation at 350 nm and photodetachment at 266.7 nm (grey). The spectrum was fit with one Gaussian (blue) and the red line is the retrieved photoelectron spectrum. As expected due to the total photon energy, this spectrum is shifted to higher eKEs than the one-colour photoelectron spectrum in Figure S6j which was recorded following photoexcitation at 310 nm and photodetachment at 310 nm.

S6. $2h\nu$ and $3h\nu$ Electron Binding Energies

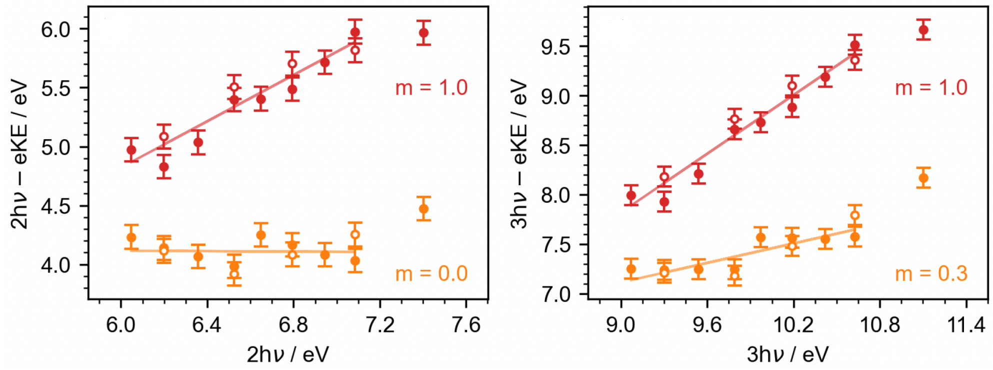

Figure S12: Plots of $nh\nu$ − eKE against $nh\nu$ for $n = 2$ (left) and $n = 3$ (right). Red and orange circles represent the areas of the middle and high eKE peaks in Figures 3 (open circles) and S6 (filled circles). The linear fits to the data recorded with wavelengths ≤ 350 nm and their slopes are also indicated.

S7. Fluorescence Spectrum

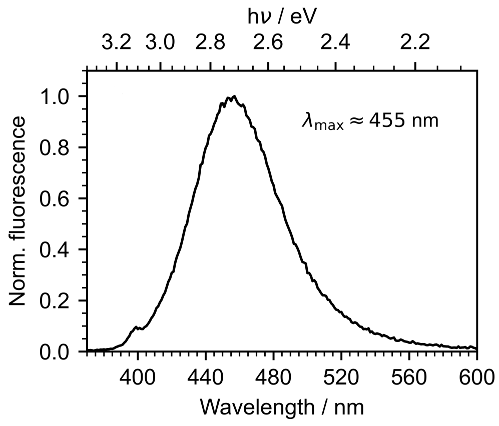

Figure S13: Normalised fluorescence spectrum of 50 µM pCE in 2.0 mM aqueous NaOH after excitation at 350 nm. The peak maximum at ∼455 nm corresponds to ∼2.72 eV.

References

(S1) Kurahashi, N.; Karashima, S.; Tang, Y.; Horio, T.; Abulimiti, B.; Suzuki, Y.-I.; Ogi, Y.; Oura, M.; Suzuki, T. Photoelectron spectroscopy of aqueous solutions: Streaming potentials of NaX (X = Cl, Br, and I) solutions and electron binding energies of liquid water and X-. *The Journal of Chemical Physics* **2014**, *140*, 174506.

(S2) Boichenko, A. N.; Bochenkova, A. V. Accurate Vertical Electron Detachment Energies and Multiphoton Resonant Photoelectron Spectra of Biochromophore Anions in Aqueous Solution. *Journal of Chemical Theory and Computation* **2023**, *19*, 4088–4099.

(S3) Thürmer, S.; Malerz, S.; Trinter, F.; Hergenhahn, U.; Lee, C.; Neumark, D. M.; Meijer, G.; Winter, B.; Wilkinson, I. Accurate vertical ionization energy and work function determinations of liquid water and aqueous solutions. *Chemical Science* **2021**, *12*, 10558–10582.

(S4) Dupuy, R.; Buttersack, T.; Trinter, F.; Richter, C.; Gholami, S.; Björneholm, O.; Hergenhahn, U.; Winter, B.; Bluhm, H. The solvation shell probed by resonant intermolecular Coulombic decay. *Nature Communications* **2024**, *15*, 6926.

(S5) Eastman, P.; Swails, J.; Chodera, J. D.; McGibbon, R. T.; Zhao, Y.; Beauchamp, K. A.; Wang, L.-P.; Simmonett, A. C.; Harrigan, M. P.; Stern, C. D.; Wiewiora, R. P.; Brooks, B. R.; Pande, V. S. OpenMM 7: Rapid development of high performance algorithms for molecular dynamics. *PLOS Computational Biology* **2017**, *13*, e1005659.

(S6) Malde, A. K.; Zuo, L.; Breeze, M.; Stroet, M.; Poger, D.; Nair, P. C.; Oostenbrink, C.; Mark, A. E. An Automated Force Field Topology Builder (ATB) and Repository: Version 1.0. *Journal of Chemical Theory and Computation* **2011**, *7*, 4026–4037.

(S7) Stroet, M.; Caron, B.; Visscher, K. M.; Geerke, D. P.; Malde, A. K.; Mark, A. E. Automated Topology Builder Version 3.0: Prediction of Solvation Free Enthalpies in Water and Hexane. *Journal of Chemical Theory and Computation* **2018**, *14*, 5834–5845.

(S8) Mobley, D. L.; Bannan, C. C.; Rizzi, A.; Bayly, C. I.; Chodera, J. D.; Lim, V. T.; Lim, N. M.; Beauchamp, K. A.; Slochower, D. R.; Shirts, M. R.; Gilson, M. K.; Eastman, P. K. Escaping Atom Types in Force Fields Using Direct Chemical Perception. *Journal of Chemical Theory and Computation* **2018**, *14*, 6076–6092.

(S9) Jorgensen, W. L.; Chandrasekhar, J.; Madura, J. D.; Impey, R. W.; Klein, M. L. Comparison of simple potential functions for simulating liquid water. *The Journal of Chemical Physics* **1983**, *79*, 926–935.

(S10) Zhang, Z.; Liu, X.; Yan, K.; Tuckerman, M. E.; Liu, J. Unified Efficient Thermostat Scheme for the Canonical Ensemble with Holonomic or Isokinetic Constraints via Molecular Dynamics. *The Journal of Physical Chemistry A* **2019**, *123*, 6056–6079.
